# Influence of Time Intervals between Bleaching Procedures on Enamel Microhardness and Surface Roughness

**DOI:** 10.2174/1874210601812010555

**Published:** 2018-07-13

**Authors:** Roberta Pimentel de Oliveira, Juliana Costa Pereira Baia, Mara Eliane Soares Ribeiro, Mario Honorato da Silva e Souza Junior, Sandro Cordeiro Loretto

**Affiliations:** Faculty of Dentistry, Federal University of Para, Augusto Corrêa Street, number 1. Guamá, Belém, Para, Brazil

**Keywords:** Dental bleaching, Bleaching agents, Peroxides, Hydrogen peroxide, Dental enamel, Hardness tests

## Abstract

**Background:**

Dental bleaching has been increasingly sought out to improve dental aesthetics, but it may cause changes in dental enamel.

**Objective:**

To assess the influence of different time intervals on bleaching gel applications with regard to the Microhardness (MH) and Surface Roughness (SR) on dental enamel.

**Material and Methods:**

Forty bovine incisors were randomly divided into two groups (G1 and G2) and both were bleached with 35% Hydrogen Peroxide (HP35) (n=20; G1: seven-day interval and G2: two-day interval). The MH and SR tests were performed before bleaching (T0) and after the first (T1), second (T2), and third (T3) bleaching gel applications. The specimens were stored in artificial saliva between each application (37°C). The data were analyzed using the student’s t-test (*p* ≤ 0.05) for dependent samples.

**Results:**

The reduced time interval (two days) led to a significant reduction in MH, while MH was not affected during the seven-day interval. The SR results increased regardless of the bleaching gel application time interval.

**Conclusion:**

The decreased time interval of two days between bleaching gel applications caused changes in MH but did not influence the SR of dental enamel.

## 1 INTRODUCTION

Nowadays, dental bleaching is one of the most popular esthetic treatments in dentistry due to its fast satisfactory results and safety when performed by a trained professional. High-concentration peroxide gels (35-38%) were developed to be used in office, especially when home bleaching is not the treatment of choice. Hydrogen peroxide acts as an oxidative agent breaking carbon double bonds present in dark pigments into hydroxyl groups, which have a lighter appearance. This reaction goes deep through the organic enamel matrix and affects its morphology [1, 2].

Several *in vitro* and *in vivo* studies have been carried out to detect possible damages to the dental substrates after bleaching treatments. The oxidizing agents present in bleaching gels are able to modify the chemical composition of the enamel, reducing concentrations of important components, such as calcium, phosphate, and fluoride [3, 4]. The reduction of enamel Microhardness (MH) detected after bleaching may be related to mineral loss, which modifies the morphology of the superficial enamel rods [5].

The Surface Roughness (SR) is also increased after bleaching treatment, which may benefit bacterial growth and pigment adherence, especially when the post-bleaching polishing is not done properly [4, 6]. These changes of the enamel seem to be strictly related to the peroxide concentration and exposure time. When these parameters are increased, the oxidation process is prolonged and intense, which may exacerbate the mentioned effects [7, 8].

Indeed, strictly related to the peroxide concentration and exposure time is the dental sensitivity. Normally, this consequence ends up to 48 hours of the gel application. Thus, during bleaching appointments, to interrupt the treatment for one week, to alleviate dental sensitivity, would be reasonable, once two or three appointments are generally necessary [9, 10]. Unfortunately, there are no scientific data to support the decision to wait a week between appointments when dental sensitivity is a concern [11]. On the other hand, a study conducted by De Paula *et al*. (2015) evaluated the effects of the reduction of the time interval between appointments to two days, and no differences in inflammatory reaction and dental sensitivity were observed when 35% Hydrogen Peroxide (HP35) was used. Indeed, the color stability observed for the two time intervals (two and seven days) remained the same.

However, regarding MH and SR, no studies were found when the time interval was shortened. Therefore, the aim of this *in vitro* study is to assess the influence of different time intervals (two and seven days) on the MH and SR after bleaching with HP35.

## 2 MATERIALS AND METHODS

This study was analyzed and approved by the Animal Ethics Committee (no. 1286260317). Medial transverse sections (10-mm high) of 40 bovine incisors were used to produce the test enamel surfaces, which were wet ground on a polishing machine (AROPOL-E, Arotec, Cotia, São Paulo) using 600, 800, 1200, and 2000 silicon carbide grit discs. The samples were randomly allocated into two groups (n=20): G1: seven-day interval, and G2: two-day interval between bleaching gel applications.

The bleaching agent used was HP35 gel (Whiteness HP Blue, FGM, Santa Catarina, Brazil). Here, 0.02 mL of the gel was applied to enamel surfaces for 40 minutes, according to the manufacturer’s instructions. During the gel application the samples were stored at 37°C. Each five minutes, the gel was disturbed in order to remove air bubbles. After bleaching procedures, samples were washed using air/water spray positioned 5 cm away for one minute and stored at 37°C in Artificial Saliva (AS) for the appropriate time interval (two or seven days).These procedures were repeated three times.

The MH and SR readings were taken before bleaching/negative control (T0) and after the first (T1), after the second (T2), and after the third (T3) bleaching gel applications. The Knoop MH tests were performed in an MH machine (Future Tech FM 700; Future Tech Interprise, Holdbrook, AZ, USA). Five readings were taken at a distance of 500 microns from each other using a 25-g load for 20 seconds each. For SR procedures, a rugosimeter (SJ-301; Mitutoyo, Los Angeles, CA, USA) was used, and the mean of three readings was taken with a track limit (Tl) of 5 mm and a cut-off (La) of 0.25 mm.

The student’s t-test for dependent samples (intragroup comparisons) was applied to the data (*P*≤ 0.05). Mean and Standard Deviation (SD) are shown in Table **1**.

## 3 RESULTS

The Highest Mean MH observed was in G1 (T0) (293.75 KHN), and the lowest was in G2 (T2) (205.08 KHN). The highest mean SR observed was in G2 (T3) (0.8141), and the lowest was in G1 (T0) (0.2433). In G1, there were no statistical differences for MH related to the number of bleaching gel applications. In G2, the reduction in the time interval between bleaching gel applications was responsible for a reduction in MH for all of the evaluated periods. There was an increase in SR values for both groups (G1 and G2) according to the number of bleaching gel applications (Tables **1** and **2**).

Different uppercase letters, *p* ≤ 0.05. Different lowercase letters, *p*≤0.05.

Therefore, as one can verify, the reduction of the time interval between appointments from seven to two days caused a significant decrease in the enamel MH, while the SR increased, regardless of the time interval between appointments.

## 4 DISCUSSION

Studies [4, 12, 13] partially disagree with the results of the present study for a seven-day interval between bleaching gel applications using HP35. Mondelli *et al*. [13] reported a trend in MH reduction, regardless of the concentration of hydrogen peroxide used. However, the MH values returned to the initial levels after a one-week remineralization period. Therefore, an explanation for the unchanged MH values in G1 was the remineralization action of the AS during the seven-day storage period between bleaching gel applications. On the other hand, when the two-day interval was considered (G2), the MH behavior was different, probably due to the short storage time.

Salomão *et al*. [12] evaluated different bleaching regimes and fluoride exposure and observed that the use of high hydrogen peroxide concentrations, similar to the one used in this study, increased the demineralization susceptibility of enamel. To minimize this effect, daily use of fluoride during bleaching procedures was suggested.

The reviewed literature shows that the addition of fluoride and calcium to bleaching agents reduces the erosion susceptibility of enamel and caries [14, 15] without compromising the bleaching efficiency [16, 17]. Therefore, the presence of calcium in the composition of the gel used in this study may have contributed to the conservation of MH values in G1.

Abouassi *et al*. [18] also used a two-day interval between bleaching gel applications, with 10% and 35% carbamide peroxide and 3.6% and 10% hydrogen peroxide gels. The values showed no MH variation regardless of the gel composition and concentration, and they were different from the results observed in the present study, probably due to differences in the gel composition.

Sasaki [19] also demonstrated that 10% carbamide peroxide and 7.5% hydrogen peroxide applied for 21 days did not alter the MH behavior; however, changes in the enamel micromorphology were detected. These results may lead to the conclusion that more expressive changes could be restricted to the use of high-concentration peroxide gels. Therefore, the enamel changes provoked during bleaching treatments seem to be more closely related to the exposure time and gel concentration. The explanation for this may be the prolonged oxidation process, which increases the effects on the enamel surface [7, 8].

An increase in the enamel SR was observed in this study, regardless of the time intervals. However, these results did not agree with others, whose SR values were not influenced by the bleaching procedures when HP35 was used [4, 20-22]. Over a two-day interval, Abouassi *et al*. made three bleaching applications and revealed an increasing trend in the SR when 10% carbamide peroxide, 35% carbamide peroxide, 3.6% hydrogen peroxide, and 10% hydrogen peroxide were used, showing that the time interval may influence SR, even when low-concentration peroxide gels were applied to the enamel surface [18].

No surface stress was applied to enamel surfaces between bleaching gel applications, which is an important aspect to mention. Not only the rubbing action of toothbrushes could influence the enamel SR, but also the presence of some compounds in toothpastes, such as detergents, fluorides, flavored substances, and abrasives. One may speculate that acidic substances present in the daily diet, associated with the action of bleaching gels, may also have an influence on the enamel surface. Dental erosion has been associated with the ingestion of sodas, energy drinks, and fruit juices [23]. Thus, the combination of bleaching agents and acidic substances present in the diet may exacerbate the negative effects on the surface of the enamel.

## CONCLUSION

The reduction in the time interval between bleaching gel applications led to a reduction in MH, while the SR values were increased regardless of the time interval.

## ETHICS APPROVAL AND CONSENT TO PARTICIPATE

This study was analyzed and approved by the Animal Ethics Committee (no. 1286260317).

## HUMAN AND ANIMAL RIGHTS

No humans were used for studies that are the basis of this research.

## CONSENT FOR PUBLICATION

Not applicable

## Figures and Tables

**Table 1:** MH descriptive statistics of different time intervals between bleaching gel applications Knoop Hardness Number (KHN).

–	G1 (N = 20)				G2 (N = 20)			
Mean	T0	T1	T2	T3	T0	T1	T2	T3
	293.751^A^	268.740^A^	265.042^A^	273.139^A^	284.523^a^	243.128^b^	205.080^c^	238.320^d,b^
	±80.76	±105.80	±68.96	±110.90	±43.91	±49.44	±56.58	±56.98

**Table 2:** SR descriptive statistics in different time intervals between bleaching gel applications.

–	G1 (N = 20)				G2 (N = 20)			
Mean	T0	T1	T2	T3	T0	T1	T2	T3
	0.2433^A^	0.3883^B^	0.4699^C^	0.5756^D^	0.3943^a^	0.6480^b^	0.7169^c,b^	0.8141^d,c^
	±0.1025	±0.1545	±0.1709	±0.2178	±0.1410	±0.2978	±0.3017	±0.3438
